# The shared biomarkers and molecular mechanisms of systemic lupus erythematosus and type 2 diabetes

**DOI:** 10.1371/journal.pone.0340312

**Published:** 2026-01-22

**Authors:** Dawei Yang, Youqi Zhang, Liu Ji, Jianjun Wu, Fan Yang

**Affiliations:** 1 Department of Orthopedics, The Fourth Affiliated Hospital of Harbin Medical University, Harbin, China; 2 Department of Cardiology, The Second Affiliated Hospital of Harbin Medical University, Harbin, China; Independent Medical Researcher and Writer, UNITED KINGDOM OF GREAT BRITAIN AND NORTHERN IRELAND

## Abstract

Systemic lupus erythematosus (SLE) and type 2 diabetes mellitus (T2DM) share inflammatory and metabolic disturbances, yet the molecular mechanism of their overlap remains unclear. This study used integrated bioinformatics to identify transcriptomic signatures and potential biomarkers common to both conditions. Gene expression profiles from publicly available datasets (SLE: 38 patients/32 controls; T2DM: 6 patients/6 controls; validation cohorts: 79/30 and 41/15, respectively) were analyzed to detect shared differentially expressed genes and co-expression modules. Functional enrichment, protein-protein interaction networks, and immune-cell composition analyses were performed. Diagnostic gene panels were constructed using random forest feature selection and logistic regression and evaluated through receiver operating characteristic analysis with external validation. A total of 551 shared differentially expressed genes were identified, enriched predominantly in type I interferon signaling, Toll-like/NOD receptor pathways, TNF signaling, necroptosis, and neutrophil extracellular trap formation. Across analytical methods, a 10-gene interferon-related hub (STAT1, IRF7, OAS1, OAS2, ISG15, MX2, IFI35, RSAD2, SAMD9, SAMD9L) genes demonstrated strong discriminative performance in both SLE and T2DM. A three-gene model further showed potential clinical utility (AUC 0.872–1.00 in discovery; 0.665–0.928 in validation).These signatures align with therapeutic axes in SLE (IFN/JAK-STAT) and intersect inflammatory-metabolic pathways in T2DM, supporting assayable biomarkers and compact diagnostic models that warrant validation in larger, medication-annotated cohorts.

## Introduction

Autoimmune diseases-exemplified by systemic lupus erythematosus (SLE)-are rising contributors to chronic morbidity worldwide, with risk and severity increasingly shaped by contemporary exposures such as dietary and microbiome shifts, xenobiotics, air pollution, psychosocial stress, and mental health conditions [1]. Although therapeutic options have expanded-illustrated by the approval of belimumab in 2011-SLE continues to confer substantial mortality from infection, lupus nephritis, neuropsychiatric complications, and cardiovascular disease; importantly, the mechanisms of SLE remain only partly understood [2].

Metabolic diseases are major drivers of global health loss; type 2 diabetes mellitus (T2DM), accounts for about 90% of diabetes cases and is projected to affect more than 640 million individuals worldwide by 2030 [3–6]. Epidemiological evidence indicates that about 10% of individuals with SLE develop T2DM within five years, underscoring the clinical relevance of their intersection [7]. Patients with concurrent SLE and T2DM frequently exhibit overlapping manifestations that are amplified by autoimmune inflammation, glucocorticoid exposure, and broader metabolic dysregulation [8,9]. For example, cohort data in adults with SLE reported diabetes in 1.9% of patients, distributed across type 1, type 2, and glucocorticoid-induced diabetes, highlighting the need for vigilant metabolic monitoring, particularly in those receiving steroids [10,11].

Despite these observations, the molecular crosstalk linking SLE and T2DM is not fully delineated. Prior work has implicated pathways such as TNF-α–mediated insulin resistance and systemic inflammatory signaling; however, the specific gene networks that converge across both conditions remain inadequately defined, and clinically actionable biomarkers are limited [12–14]. Current therapeutic approaches in SLE frequently encounter limitations related to efficacy and adverse effects, emphasizing the necessity for novel candidate biomarkers for risk stratification and therapeutic targets [15,16].

Advances in bioinformatics and high-throughput sequencing now enable deeper exploration into genetic and molecular intersections between complex diseases [17]. Thus, we sought to interrogate the shared transcriptomic architecture of SLE and T2DM using integrated bioinformatics across discovery and validation cohorts. Our objectives were threefold: (i) to identify differentially expressed genes common to both diseases and characterize their functional enrichment; (ii) to define co-expression modules and protein-protein interaction hubs that capture the core immunometabolic signal; and (iii) to derive and externally evaluate parsimonious diagnostic models based on shared gene signatures. Our findings provide novel insights into common pathogenic pathways and immune dysfunction, establishing a foundation for precision diagnosis and targeted therapy of these complex diseases.

## Materials and methods

### Study design and data collection

**Fig 1:**
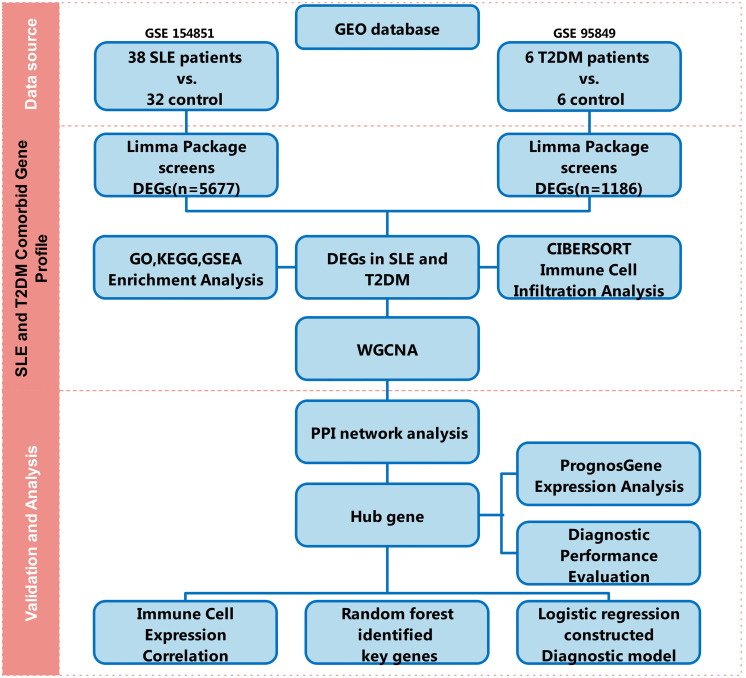
Overview of the analysis workflow. Gene expression datasets for SLE (GSE154851) and T2DM (GSE95849) were obtained from the GEO database. Differentially expressed genes (DEGs) were identified using the limma package. Shared DEGs underwent enrichment analysis (GO/KEGG), immune infiltration analysis (CIBERSORT), and co-expression network analysis (WGCNA). Random forest and logistic regression was used for feature selection, and multivariable logistic regression was used to construct diagnostic models. Candidate genes/models were evaluated by expression differences and ROC analysis, and further explored using PPI networks and immune-cell association analyses.

This was a bioinformatics study based on a comparative analysis of publicly available transcriptomic datasets. GEO records were queried in the GEO DataSets interface (targeting GEO Series, GSE, rather than GEO Profiles) using the keywords ‘systemic lupus erythematosus’ OR ‘SLE’, ‘type 2 diabetes’, ‘human’, and ‘expression profiling by array’. Candidate series were screened and included if they (i) were human microarray studies, (ii) contained clearly annotated case-control groups, and (iii) provided de-identified expression data suitable for downstream re-analysis. The final eligible series comprised discovery datasets GSE154851 (whole/peripheral blood: 38 SLE vs 32 controls) and GSE95849 (peripheral blood; GPL22448: 6 T2DM vs 6 controls), and validation datasets GSE61635 (SLE: 79 vs 30) and GSE250283 (T2DM: 41 vs 15). To avoid pseudo-replication due to repeated measurements, we retained only the first visit sample for each patient. For data acquisition, series matrix files and platform annotations were downloaded programmatically in R using the GEOquery package (getGEO; GSEMatrix=TRUE), and sample phenotypes (case/control) were extracted from GEO sample metadata. SLE classification was according to the 1982 ARA revised criteria (updated by the ACR in 1997) as reported by the originating studies, and T2DM diagnosis was based on ADA diagnostic criteria as reported by the originating studies (Standards of Care updated annually). Medication metadata (including glucocorticoid exposure) were not available in these GEO records; therefore, glucocorticoid-associated dysglycemia could not be excluded among diabetes cases. Dataset details with methodological steps illustrated in Fig 1 and Table 1.

**Table 1:** Characteristics of GEO microarray datasets in discovery and validation phases.

Disease	GEO ID	Phase	Tissue Source	Experiment Type	Case vs. Control^1^
SLE	GSE 154851	Discovery	Peripheral blood	Expression profiling by array	38 *vs.* 32
	GSE 61635	Validation	Peripheral blood	Expression profiling by array	79 *vs.* 30
T2DM	GSE 95849	Discovery	Peripheral blood	Expression profiling by array	6 *vs.* 6
	GSE 250283	Validation	PBMCs	Expression profiling by array	41 *vs.* 15

^1^Case vs. control indicates the number of disease samples versus healthy controls in each dataset.

### Differential gene expression analysis

Differential gene expression analysis was performed to quantify genes statistically significantly upregulated or downregulated in SLE and T2DM compared with controls [18]. Differential expression contrasted cases versus controls within each dataset. Unless otherwise specified, significance was defined as FDR (Benjamini-Hochberg) <0.05 with |log2FC| ≥ 1 [19]. Volcano plots display log2FC versus -log10(P). The SLE and T2DM DEG lists were intersected to obtain the shared set for downstream analysis. No explicit batch-effect correction was applied prior to differential expression analysis because batch covariates were not consistently available in GEO metadata and datasets were analyzed separately without cross-study merging.

### Weighted gene co-expression network analysis (WGCNA)

Weighted gene co-expression network analysis (WGCNA) was conducted to identify disease-associated co-expression modules and to prioritize network-level candidate genes beyond single-gene differential expression. Networks were built separately in SLE and T2DM, modules were defined by dynamic tree cutting, and module-trait correlations were computed to nominate modules significantly associated with disease status [20].The gene lists from these significant modules in each disease were then intersected to derive candidate shared key genes.

### Protein-protein interaction (PPI) networks

Protein-protein interaction (PPI) network analysis was used to contextualize shared candidate genes in curated interaction space and to prioritize hub genes based on network topology. Interactions were queried from STRING and visualized in Cytoscape; hub genes were ranked using cytoHubba across complementary centrality metrics [21]. Hub genes were nominated by topological ranking (e.g., MCC/degree/betweenness in cytoHubba); the intersection of top-ranked lists across algorithms yielded the 10-gene interferon-centered hub (STAT1, IRF7, OAS1, OAS2, ISG15, MX2, IFI35, RSAD2, SAMD9, SAMD9L) [22,23]. Heatmaps display z-scored expression.

### Functional enrichment analysis

Functional enrichment analysis was conducted to interpret biological processes and pathways over-represented among shared DEGs and module-derived genes, and to quantify coordinated pathway-level shifts across the ranked transcriptome. GO (BP/CC/MF) and KEGG over-representation analyses were performed in R using clusterProfiler, with hypergeometric testing and Benjamini-Hochberg adjustment (FDR < 0.05). Pre-ranked GSEA was applied to the full gene list ordered by log2 fold change to assess pathway enrichment between cases and controls. Enrichment results were identified from the ranked outputs, and were then thematically grouped (e.g., immune/inflammatory, metabolic, and signaling processes) for narrative synthesis and for comparing shared versus disease-specific patterns between SLE and T2DM.

### Predictive modeling and validation

We developed gene-expression–based prediction models to estimate the probability under the reference labels provided by the original GEO studies. Because SLE lacks a single diagnostic gold standard and GEO annotations reflect study-defined clinical/classification ascertainment rather than a universal reference standard, model performance was interpreted as discrimination for case-status prediction rather than diagnostic accuracy. Discrimination was quantified using ROC curves and the area under the curve (AUC/c-statistic), which represents the probability that a randomly selected case is assigned a higher predicted score than a randomly selected control. Model calibration was evaluated using bootstrap resampling and calibration curves as previously described. Feature ranking was conducted using random forests, followed by logistic-regression model fitting in discovery cohorts and bvalidation in independent datasets. A logistic-regression classifier (glm, binomial link) was fit on the discovery cohort using the selected genes. Model discrimination was evaluated by ROC curves and AUC (DeLong 95% CIs; pROC). Decision thresholds were explored by the Youden index. We applied bootstrap resampling (B = 1,000) to estimate optimism-corrected performance and to draw calibration curves (rms package; apparent vs bias-corrected). The trained model and fixed coefficients from discovery were applied-after per-gene z-scaling within each dataset-to independent cohort (GSE61635 for SLE; GSE250283 for T2DM). ROC/AUC and calibration were re-computed. No re-training was performed in the test sets to preserve a locked-model external validation framework, thereby providing an unbiased estimate of transportability and avoiding information leakage and optimism that would arise from refitting on validation cohorts.

### Statistical considerations

All tests reported in this study-including those used for differential expression, enrichment analyses, correlation-based analyses, and ROC/AUC inference as specified-were two-sided. Multiple testing was controlled by the BH procedure unless stated otherwise. For visualization, expression values were center-scaled by gene (z-score). Missing values, if present, were handled by listwise deletion for correlation and by complete-case analysis for modeling (sensitivity checks confirmed stability). Reproducible code was implemented in R (v4.x) using limma, clusterProfiler, WGCNA, STRING/cytoHubba (via Cytoscape), CIBERSORT, randomForest, pROC, and rms.

### Ethics

All analyses were performed on fully anonymized, pre-existing datasets; no new human subjects were recruited. In accordance with the Declaration of Helsinki [24], institutional review board (IRB) approval and informed consent were not required for this secondary data analysis. This study involved only secondary analysis of de-identified, publicly available microarray data. No patients, relatives, or public representatives were directly involved in the design, conduct, or dissemination of this research. Participant-level ancestry/ethnicity information was not consistently available in the GEO records and was not analyzed in this secondary study. All data handling complied with relevant data-protection and patient-privacy regulations.

## Results

### Identification of 551 shared differentially expressed genes between SLE and T2DM

**Fig 2:**
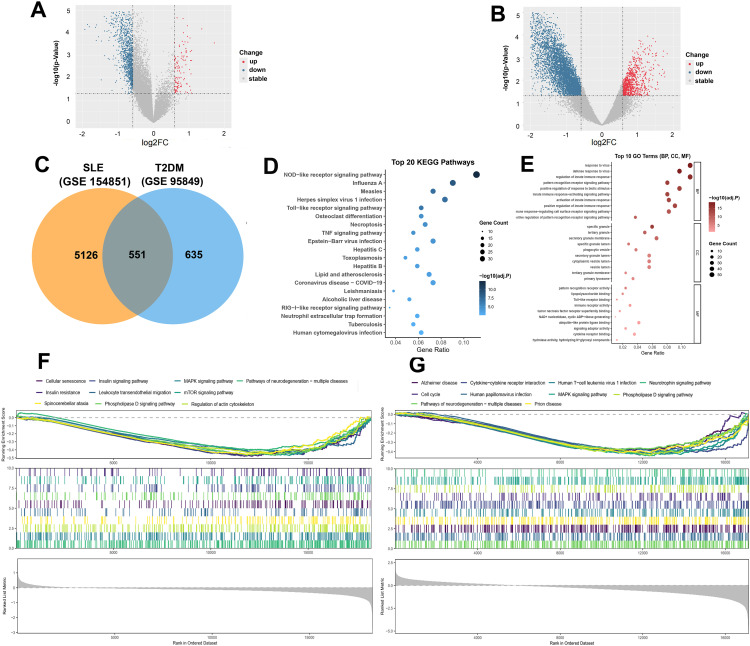
Differential expression and enrichment. **(A)** Volcano plot, SLE (GSE154851). **(B)** Volcano plot, T2DM (GSE95849). **(C)** Venn diagram of shared/specific DEGs. **(D)** KEGG enrichment of shared DEGs. **(E)** GO enrichment of shared DEGs. **(F)** Preranked GSEA in SLE**. (G)** Preranked GSEA in T2DM.

Volcano plots for SLE (GSE154851, Fig 2A) and T2DM (GSE95849, Fig 2B) showed extensive up- and down-regulation. Intersection analysis identified 551 shared DEGs, alongside 5,126 SLE-specific and 635 T2DM-specific genes (Fig 2C). The shared DEG set was dominated by innate immune and inflammatory programs (pattern-recognition receptor signaling, cytokine/TNF-related signaling, cell-death modules, and NET-related pathways), while cardiometabolic signals included KEGG ‘Lipid and atherosclerosis’ and additional metabolism/redox- and endocrine-related pathways (Fig 2D–2G).

### Identification of an IFN-centered 10-gene hub shared by SLE and T2DM

**Fig 3:**
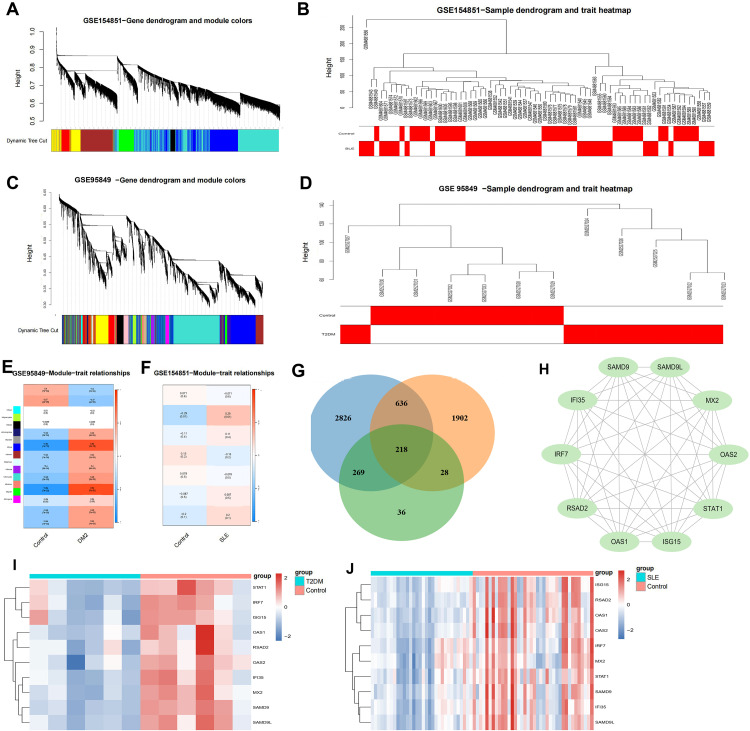
Co-expression modules and hub nomination. **(A)** Gene dendrogram and module colors in T2DM (WGCNA). **(B)** Sample clustering dendrogram and case/control trait heatmap in T2DM. **(C)** Gene dendrogram and module colors in SLE. **(D)** Sample clustering dendrogram and case/control trait heatmap in SLE. **(E-F)** Module-trait relationships in T2DM and SLE, respectively. **(G)** Intersection of disease-associated modules with shared DEGs. **(H)** PPI network of intersected genes and hub-gene ranking. **(I-J)** Expression patterns of the 10 hub genes in T2DM and SLE discovery cohorts.

Using WGCNA as an integrative framework, we distilled thousands of disease-responsive genes into a reproducible interferon-centered hub shared by SLE and T2DM (Fig 3A–3F). Sample clustering did not reveal outliers and disease labels aligned with the top dendrogram splits (Fig 3B, 3D), indicating stable network construction. Dynamic tree cutting identified multiple modules per dataset (Fig 3A, 3C). PPI-centricity and module connectivity prioritized a 10-gene hub panel-IFI35, IRF7, ISG15, MX2, OAS1, OAS2, RSAD2, SAMD9, SAMD9L, STAT1-that consistently separated cases from controls (Fig 3H–3J).

### Predictive modeling and external validation of the IFN 10-gene Hub

**Fig 4:**
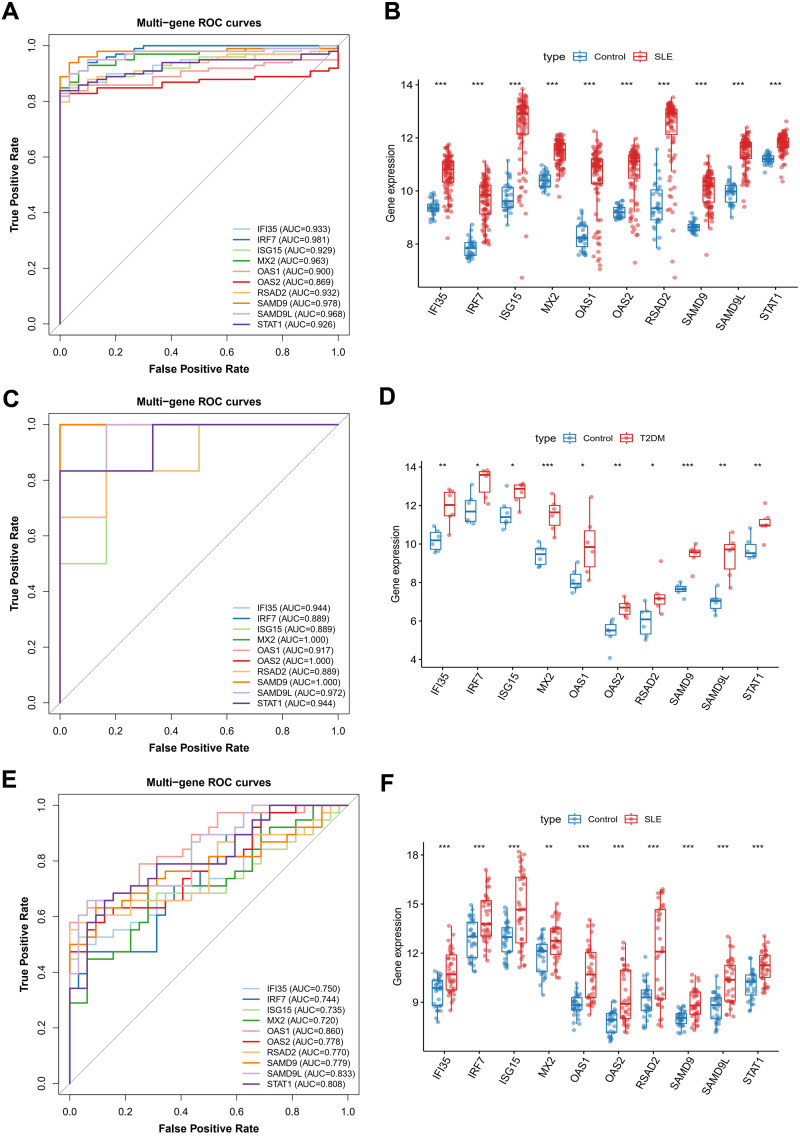
Diagnostic performance and external validation. **(A)** ROC curves, SLE discovery. **(B)** Expression of 10 hub genes, SLE discovery. **(C)** ROC curves, T2DM discovery. **(D)** Expression of 10 hub genes, T2DM discovery. **(E)** ROC curves, SLE validation. **(F)** Expression of 10 hub genes, SLE validation. Box plot: X-axis indicates gene, Y-axis indicates expression level. The t-test was used to compare the means of the two groups (* indicates p < 0.05, ** indicates p < 0.01, *** indicates p < 0.001).

We next assessed the discriminative performance of the 10-gene interferon-centered hub (IFI35, IRF7, ISG15, MX2, OAS1, OAS2, RSAD2, SAMD9, SAMD9L, STAT1) [23]. In the discovery SLE cohort, single-gene ROC curves showed uniformly high accuracy with AUCs ranging from 0.86 to 0.98 (Fig 4A). Concordantly, expression levels were markedly elevated in cases versus controls for all ten genes (Fig 4B; P < 0.001), indicating a coherent ‘IFN-high’ signature. Notably, while interferon-related genes beyond type I (including interferon type II and type II-associated transcripts) were observed among the broader set of differentially expressed genes, only type I interferon-linked genes retained high network centrality and cross-disease robustness and therefore converged as hub genes in the integrated network framework.

In the discovery T2DM cohort, the same hub remained strongly discriminative with AUC 0.89–1.00 (Fig 4C), and all genes were significantly up-regulated in patients (Fig 4D). Notably, several genes (e.g., MX2, OAS2, SAMD9) approached 1.00, reflecting near-perfect separation in this dataset.

External validation in an independent SLE dataset yielded consistent but attenuated performance (AUC 0.71–0.86, Fig 4E) with persistent case-control over-expression (Fig 4F; P < 0.001).

### Immune cell lineage interaction with IFN 10-gene Hub

**Fig 5:**
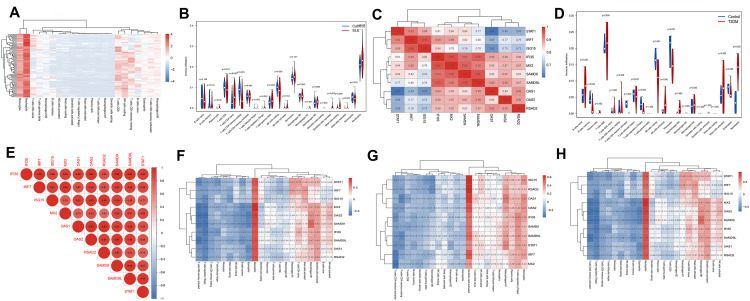
Immune infiltration and hub–cell associations. **(A-B)** Immune fractions and group differences, SLE. **(C)** Hub-gene correlation matrix, SLE. **(D)** Immune fractions and group differences, T2DM. **(E)** Hub-gene correlation matrix, T2DM. **(F-H)** Correlations between hub genes and immune fractions.

In SLE, hierarchical clustering of the CIBERSORT-derived fractions separated most cases from controls (Fig 5A), and multiple compartments differed significantly between groups (Fig 5B; P values annotated). T2DM exhibited a concordant though less pronounced redistribution of immune subsets (Fig 5D), indicating that both diseases share a shift towards an innate/inflammatory milieu.

The 10-gene hub formed a tightly co-expressed block in both cohorts (pairwise r predominantly ≥0.8; Fig 5C, 5E), consistent with a concerted interferon program rather than isolated gene changes. Correlation heatmaps linking hub genes with immune fractions showed a reproducible pattern across datasets (Fig 5F–5H): hub expression tracked positively with innate immune readouts-including dendritic cell (activated dendritic cells) and macrophage lineages (M0/M1 macrophages) and negatively with naive/resting lymphocyte pools.Here, we summarized all CIBERSORT-inferred peripheral immune-cell subsets (innate vs adaptive; circulating vs potentially tissue-migrated phenotypes) and their correlations with the 10-gene hub in Table 2.These cell-level couplings align with an interferon-high endotype characterized by antigen presentation and inflammatory activation, providing a mechanistic bridge between the transcriptomic signature (Figs 2–4) and the observed immunophenotype.

**Table 2:** Summary of immune cell categories and their associations.

Immune Cell Type	Innate/Adaptive	Compartment Category^1^	Association With Hub Genes	Notes
**Monocytes**	Innate immunity	Peripheral immune cells	Positive association with STAT1, IRF7, OAS family, ISG	Central mediators of IFN-driveninflammatory activation
**Macrophages**	Innate immunity	Peripheral and potentiallytissue-migrated	Positive association, especially withISG15 and RSAD2	Reflect IFN-associated inflammatory polarization
**Dendritic cells (resting/activated)**	Innate immunity	Peripheral immune cells	Positive correlation with STAT1, IRF7,OAS1/2	Antigen-presenting cells highly responsive to IFN
**Neutrophils**	Innate immunity	Peripheral immune cells	Weak to moderate positive association	Contribute to NET-related and IFN-high immune signatures
**NK cells**	Innate immunity	Peripheral immune cells	Variable or weak correlation	IFN modulated cytotoxic function
**CD8**^**+**^ **T cells**	Adaptive immunity	Peripheral immune cells	Negative association across hub genes	Reduced naïve/cytotoxic CD8^+^T cell fractions in IFN-high
**CD4+memory T cells**	Adaptive immunity	Peripheral immune cells	Negative association	Redistribution toward innated-inflammatory axis
**Naive/memory B cells**	Adaptive immunity	Peripheral immune cells	Weak or negligible correlation	Age-associated B cells(ABCs)cannot be detected
**Plasma cells**	Adaptive immunity	Peripheral immune cells	Weak positive association	Reflect humoral activation under an IFN-skewed environment

^1^All immune-cell fractions were inferred from peripheral-blood transcriptomes. “Peripheral/ potentially tissue-migrated” indicates phenotypes that may include cells recently emigrated from tissues into the circulation, but true tissue infiltration cannot be determined from these data.

### Parsimonious diagnostic models derived from the interferon hub

**Fig 6:**
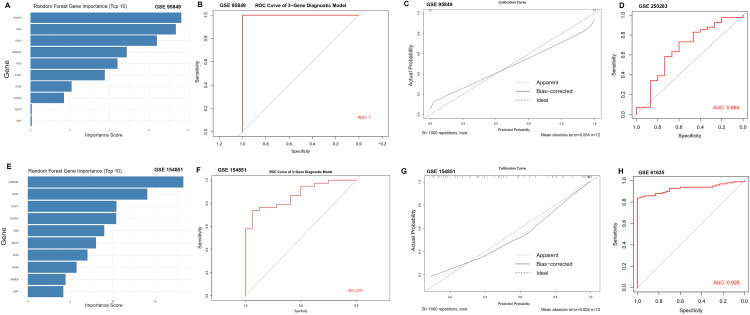
Feature selection and diagnostic modeling. **(A)** Random-forest feature importance, T2DM discovery. **(B)** ROC curve, T2DM three-gene model. **(C)** Calibration, T2DM model. **(D)** ROC curve, T2DM validation. **(E)** Random-forest feature importance, SLE discovery. **(F)** ROC curve, SLE three-gene model. **(G)** Calibration, SLE model. **(H)** ROC curve, SLE validation.

Random-forest ranking highlighted a small subset of features as dominant contributors in each discovery cohort (Fig 6A, 6E). Using the top-ranked genes, we trained a three-gene classifier that achieved excellent discrimination in discovery: AUC = 1.00 for T2DM (GSE95849; Fig 6B) and AUC = 0.872 for SLE (GSE154851; Fig 6F). Bootstrap calibration curves are shown in Fig 6C and 6G.

External testing revealed disease- and dataset-dependent generalizability. Applied to an independent T2DM dataset (GSE250283), performance was modest (AUC = 0.665; Fig 6D), whereas in an independent SLE dataset (GSE61635) it remained high (AUC = 0.928; Fig 6H).

## Discussion

Previous studies have suggested that patients with SLE exhibit immune perturbations that predispose to disordered glucose metabolism, including autoantibody-mediated interference with insulin signaling and glucocorticoid-related dysglycemia in susceptible individuals [25–27]. However, despite this clinical overlap, little is known about the cross-disease molecular programs that link autoimmunity to metabolic dysfunction which refers to insulin resistance/impaired insulin signaling, accompanied by β-cell dysfunction-related dysglycemia and atherogenic dyslipidemia/metabolic-syndrome features [28]. Clarifying these programs could facilitate risk stratification and inform precision co-management.

Our integrative analysis identified 551 shared DEGs between SLE and T2DM enriched for type I interferon (IFN-I) signaling, NOD/Toll-like receptor pathways, TNF signaling, neutrophil extracellular trap formation, and necroptosis (Fig 2C–2E). These findings accord with inflammatory axes reported in each disease. Despite this convergence, the extent to which immune activation couples to metabolic pathways has been incompletely characterized. Our GSEA adds this layer by demonstrating enrichment of insulin signaling/resistance, MAPK and mTOR pathways, leukocyte trans-endothelial migration, and cytoskeletal regulation (Fig 2F–2G), thus connecting an IFN-skewed state to metabolic homeostasis and vascular activation.

WGCNA distilled thousands of disease-responsive genes to a 10-gene interferon-centric hub that is reproducibly activated in SLE and T2DM (Fig 3A–3D). Subsequent PPI network analysis highlighted 10-gene interferon-centered hub (STAT1, IRF7, OAS1, OAS2, ISG15, MX2, IFI35, RSAD2, SAMD9, SAMD9L) [23,29,30]-each demonstrating diagnostic value across multiple validation cohorts. ROC analyses confirmed their utility as candidate biomarkers for risk stratification, exhibiting high sensitivity and specificity. Elevated expression levels of these genes in disease states further affirm their involvement in the pathological processes of SLE and T2DM (Fig 4).

RSAD2 is significantly upregulated in SLE patients compared to controls, with implications for the determination of disease activity in SLE patients and a close link in the development of SLE. Peripheral-blood transcriptomic studies consistently demonstrate elevated OAS1/OAS2 expression in SLE with correlations to disease activity [31]. Within metabolic inflammation, convergent evidence also implicates the IFN-OAS axis in T2DM: in human skin and skeletal muscle transcriptomes, OAS1/OAS2/OAS3 are significantly upregulated and enriched for infection-related pathways [32,33], indicating tissue-level activation of interferon responses in T2DM [34]. These IFN/JAK-STAT-aligned hubs complement B-cell-directed therapy (e.g., belimumab) in SLE and may delineate an immunometabolic subgroup in T2DM beyond metformin-centered metabolic control. Beyond our results, prior integrative bioinformatics analyses-similarly identified an IFN-centered pathogenic program in SLE, further supporting the robustness of an interferon-driven molecular axis across independent datasets [35]. The convergent findings reinforce the biological plausibility of the shared IFN-high signature we observed in both SLE and T2DM. Notably, the consistently higher diagnostic performance observed in the independent SLE validation cohort, compared with the T2DM validation cohort, suggests that the interferon-centered hub may be more informative for SLE identification than for type 2 diabetes. This asymmetry likely reflects the more fundamental and disease-defining role of type I interferon signaling in SLE pathophysiology, whereas interferon-related activation in T2DM may be secondary, context-dependent, or biologically heterogeneous.

Therapeutically, evidence on how immunosuppressants modulate glucose metabolism in SLE remains limited. Calcineurin inhibitors (e.g., tacrolimus, cyclosporine A) can impair insulin secretion and reduce insulin sensitivity, with animal data indicating progressive β-cell dysfunction at moderate-to-high doses [36,37]. However, hydroxychloroquine has been reported to improve insulin resistance, enhance β-cell function, increase adiponectin, and augment insulin-receptor affinity [38]. Associations between mycophenolate mofetil and steroid-induced diabetes, and case reports of cyclophosphamide-related autoimmune diabetes, further highlight a complex therapeutic landscape [39,40]. Despite these observations, robust, prospective data linking specific drug exposures, IFN-high endotypes, and glycemic outcomes are scarce. Thus, future studies should integrate longitudinal transcriptomics with detailed medication histories to identify patients most likely to benefit from targeted immunometabolic interventions.

Our study identified shared transcriptomic signatures between SLE and T2DM, highlighting a potential immuno-metabolic axis common to both diseases. Given the growing prevalence of metabolic complications in patients with autoimmune disorders, clinicians must prioritize metabolic monitoring and management strategies when prescribing treatments such as glucocorticoids or immunosuppressants.

### Limitations

Our study has several limitations that should be addressed in future research. Several limitations should be acknowledged. First, across discovery and validation datasets for both SLE and T2DM, control groups were consistently smaller than case groups, creating an unbalanced design that can inflate variance estimates and reduce power for detecting moderate, cross-disease signals. This imbalance-together with small discovery samples (notably the T2DM discovery set)-may have biased shared-gene discovery toward the strongest, most coherent programs (type I IFN/ISGs) while impeding detection of weaker but biologically relevant overlaps, including potential type II/type III interferon-related pathways. In addition, GSE95849 includes three groups-diabetic peripheral neuropathy (DPN), diabetes mellitus (DM), and healthy controls; although we restricted analyses to the DM-versus-control comparison, the presence of a neuropathy-defined subgroup and the original study design may limit generalizability to unselected T2DM populations.

Second, the study did not highlight B-cell involvement (including ABC-like compartments) or robust hub interactions with B/T lineages, which is potentially problematic in a multi-autoantibody disease such as SLE [41].

Third, participant ancestry was incompletely reported across GEO records, and at least one cohort is ancestry-specific (e.g., Filipino participants), which constrains transportability across populations and underscores the need for multi-ancestry validation.

Finally, reliance on microarray data limits precision-medicine resolution (probe dependency, dynamic range, isoforms/novel transcripts). Future work should validate these signatures using harmonized RNA-seq (ideally single-cell/spatial), longitudinal sampling, and detailed medication/comorbidity annotation.

## Conclusion

Our integrative bioinformatics analyses delineate a reproducible, interferon-anchored inflammatory program shared by SLE and T2DM and nominate a compact gene panel with independent external validation. These results support a tractable framework for immunometabolic stratification and provide a rationale for prospective, clinically deep-phenotyped multi-omics studies to confirm mechanistic relevance and clinical utility.
